# Reveals meat quality and muscle metabolism characteristics in naturally grazed Sunit sheep at different ages

**DOI:** 10.1016/j.fochx.2025.103012

**Published:** 2025-09-07

**Authors:** Lu Chen, Xige He, Yunfei Han, Yajuan Huang, Jin Li, Xueting Yu, Xueyan Yun, Yirgejim Wuqier, Gerelt Borjigin

**Affiliations:** a College of Food Science and Engineering, Inner Mongolia Agricultural University, Hohhot 010018, China; b State Key Laboratory of Reproductive Regulation and Breeding of Grassland Livestock, School of Life Sciences, Inner Mongolia University, Hohhot 010020, China; c Inner Mongolia Academy of Agricultural and Animal Husbandry Sciences, Hohhot 010031, China

**Keywords:** Ages, Meat quality, Muscle metabolism, Sunit sheep

## Abstract

This study aimed to explore the effects of different ages on muscle metabolism and its association with meat quality in naturally grazed Sunit sheep using non-targeted and targeted metabolomics. The results indicated that meat quality parameters, such as cooking loss, shearing force, and intramuscular polyunsaturated fatty acid content, were most favorable at age of 6 months (M-1) than those at 18 (M-2) and 30 months (M-3). A total of 105 metabolites were identified. The key upregulated metabolites and metabolic pathways were dominated by polyunsaturated fatty acids and glycerophospholipid metabolism in M-1, whereas the key downregulated metabolites were dominated by organic acids and their derivatives. Correlation analysis showed that lysoglycerophospholipids, citrulline, and succinic acid are tenderness-related metabolites. Dipeptides, inosine 5′-monophosphate, and guanosine 5′-monophosphate are flavor-related metabolites. These findings provide a comprehensive insight into muscle metabolism and metabolic pathways in Sunit sheep meat at different ages

## Highlights

• Polyunsaturated fatty acids and glycerophospholipid metabolism were prominent at M-1.
• Organic acids and their derivatives were downregulated at M-1.
• Lysophospholipid, citrulline, and succinic acid were tenderness-related metabolites.
• Dipeptides, inosine 5ʹ-monophosphate, and guanosine 5′-monophosphate were flavor-related metabolites.

## 1 Introduction

Animal age has always been a key factor that producers considered for economic benefits and meat quality. Consumer preferences for age vary by region, with mutton being mostly discussed. Mutton is characterized by a typical species-related flavor named “mutton flavor”. In grasslands, herders who graze for a living prefer adult mutton, especially naturally grazed mutton. Mutton provides a substantial amount of dietary protein and fat (Kang et al., 2024; Zhang et al., 2022), thereby offering considerable nutritional value, and fatty and amino acids (Bao et al., 2021; Prache et al., 2022). Unlike the meat of other ruminant animals, mutton exhibits a unique meaty aroma and flavor characteristics (Prache et al., 2022). Meat quality is a complex trait that is affected by many factors, including diet (Zhang et al., 2022), sex (Watkins et al., 2010), age (Bao et al., 2021; Prache et al., 2022), and genotype (Mohammadabadi et al., 2021).

Sheep muscle tissue undergoes stable growth with age (Prache et al., 2022). A positive correlation was found between age and meat tenderness (Bao et al., 2021). Biochemical components within the muscle tissue undergo changes with increasing age, such as the increase in intramuscular fat (IMF) content and decrease in water content (Bao et al., 2021; Prache et al., 2022). age and IMF content can indirectly affect meat color, pH, and flavor components (Prache et al., 2022). In addition, muscle proteins directly affect the growth performance and meat quality traits of animals because proteolysis and amino acid release are mainly involved in muscle metabolism (Kobayashi et al., 2019). Bao et al. (2021) analyzed the fatty acid composition of Tibetan sheep at various ages and discovered that beneficial polyunsaturated fatty acids (PUFAs) for human health decreased with increasing age. Particularly, the concentration of fatty acids contributing to the development of ‘mutton flavor’ increases with age (Watkins & Frank, 2019). Some chemical experiments have demonstrated that the concentration of flavor-affecting compounds in sheep increases with age (Chen, He, et al., 2024; Watkins et al., 2010).

Muscle metabolism in animals is also affected by age. Ma et al. (2022) found that in different developmental stages of Tan sheep, the functional status usually determines the extent to which metabolic pathways operate. Zhang et al. (2022) suggested that modifying muscle metabolism could enhance the quality and nutritional content of Tibetan sheep meat. Wang, Wang, et al. (2021) revealed the potential characteristic metabolites in muscle using metabolomics to discriminate between sheep and goat meat. Several factors can influence alterations in animal muscle metabolism, including alterations in the pre-slaughter diet (Ramírez-Zamudio et al., 2022) and rearing environments (Zhang et al., 2022). Previous studies have shown that naturally grazing sheep have more complex and diverse feeding behaviors than their barn-fed counterparts, and that this significantly affects the quality of animal products (Webb & Casey, 2010). These differences are mainly reflected in the growth performance, and meat quality of sheep. Naturally grazed sheep have high levels of amino acids and carnitine, which may contribute to enhanced functional quality of sheep meat (Wang, Xu, et al., 2021). To the best of our knowledge, there is limited research on the effect of age on muscle metabolism, particularly in naturally grazed Sunit sheep.

Sunit sheep (*Ovis aries*) are raised in the Sunit grassland, characterized by a continental climate with an annual mean temperature of 4.3 °C and extreme seasonal variations (ranging from −38.8 °C to 38.7 °C) (Gao et al., 2023). Where they feed exclusively on wild grass in their natural habitat. The wild grasses they primarily consume include species from the genus *Stipa*, *Achnatherum splendens*, the genus *Caragana*, the genus *Artemisia*, and *Allium* plants. In which *Allium mongolicum Regel*, a typical herb of the *Allium* family, grows extensively in the desert steppe of Inner Mongolia, Sunit Distric (Liu & Ao, 2021). *Allium* plants have been proposed as potential additives that could improve the productive performance and meat quality of animals (Samolińska et al., 2020). *Allium mongolicum regel* is rich in flavonoids, polysaccharides, essential oils, and other bioactive compounds (Li et al., 2019). Previous studies have reported that the dietary supplementation of sheep diets with flavonoids from *Allium mongolicum Regel* could promote growth performance, as well as improve meat quality and mutton flavor of lambs (Liu & Ao, 2021).

Sunit sheep meat exhibits a unique nutritional richness (Kang et al., 2024) and exquisite flavor (Zhang, Sun, et al., 2024). Our prior investigations have demonstrated that age-related variations exert a significant influence on the mechanisms underlying adipose tissue deposition in Sunit sheep (Chen, Zhang, et al., 2024). In this study, we hypothesized that variations in age would affect meat quality, fatty acid composition, amino acid composition, and muscle metabolism in Sunit sheep. Therefore, the meat quality parameters and nutrient composition of Sunit sheep meat were analyzed. Conducting qualitative analysis of the sample compounds using the ultra-high-pressure liquid chromatography-mass spectrometry (UPLC-MS) can eliminate interference from the mixture (Azooz et al., 2022), thereby determining the metabolic characteristics of muscle tissues at different ages. The distributions of amino acids and fatty acids were further targeted for verification. The purpose of this study was to reveal the muscle metabolism profile of naturally grazed Sunit sheep at different ages and provide basic metabolic data to facilitate the enhancement of sheep meat quality. This will enhance the economic benefits for the sheep meat industry and foster advancements in the field of animal husbandry.

## 2 Materials and methods

The study protocol was approved by the Ethics Committee of Inner Mongolia Agricultural University [approval document number (NND2023092)] and complied with the guidelines of the Chinese Academy of Animal Health Research (GB 14925–2010). All experimental procedures were conducted in compliance with the European Union's welfare guidelines (Directive 2010/63/EU). Sunit sheep represent a distinctive breed among the Mongolian sheep varieties. The experimental animals were provided by same herd (Boyint Ranch, Zhuruhe region) from Sunit Right Banner, Inner Mongolia Autonomous Region, and were utilized for meat quality research. All animals were transported to commercial slaughterhouses located 6.5 km away. After a 12-h rest period with access to water, they were humanely slaughtered by trained personnel using stunning followed by exsanguination (Menci et al., 2023).

### 2.1 Sample collection

Eighteen Sunit sheep castrated rams aged 6-month-old (M-1, *n* = 6; mean weight: 29.56 ± 0.87 kg), 18-month-old (M-2, n = 6; mean weight: 49.64 ± 0.53 kg), and 30-month-old (M-3, n = 6; mean weight: 57.03 ± 1.37 kg) were randomly selected. The sheep herds exhibited daily activity durations of up to 10 h (from 07:00 to 17:00), during which they had unrestricted access to water and grazed freely on natural pastures under an extensive grazing system with abundant forage species which mentioned in introduction section. After slaughter, 5 g of the longissimus thoracis et lumborum (LTL, 5th to13th ribs) was retrieved within 5 min, transferred into pre-labeled cryotubes, and subsequently preserved in liquid nitrogen to facilitate metabolite extraction and detection. Simultaneously, 200 g of muscle tissue was collected from the same anatomical location, placed in a sealed plastic bag, and stored at 4 °C for 24 h for subsequent analysis of meat quality, fatty acids, and amino acids.

### 2.2 Meat quality analysis

An automatic colorimeter (TCP2, Oi ke Photoelectric Instrument Ltd., Hefei, China) was used to determine the color of the cut surface of each sample after 1 h of blooming (4 °C), where L*, a*, and b* represent lightness, redness, and yellowness, respectively. Prior to measurement, the instrument was preheated for 15 min and calibrated using a standard white calibration plate. The pH was determined using a digital pH meter (PB-10, Shengchangda Instrument Co. Ltd., Beijing, China). Before the measurement, the pH meter was calibrated using three standard buffer solutions (pH 4.01, pH 6.86, and pH 9.18). The pH meter was inserted into the meat sample to a depth of 2 cm to measure the pH of the LTL muscle at 45 min and 24 h post-slaughter (stored at 4 °C). The recorded values were denoted as pH_45min_ and pH_24h_, respectively. Approximately 50 g of the meat sample was required to determine cooking loss. Each sample was packed in a plastic bag and cooked in a constant temperature water bath at 80 °C. It should be noted that all samples were processed in a single cooking batch. When the center temperature of the meat sample reached 70 °C, the sample was removed and filter paper was used to absorb surface moisture. The weights of the sample before (M1, g) and after cooking (M2, g) were recorded. The formula for calculating cooking loss can be expressed as (1):

(1) $$\left(M_{1}-M_{2}\right)/M_{1}✕100\%$$

Subsequently, the meat samples were cut into 1-cm^3^ cubes for shear force measurement (C-LM38, Northeast Agricultural University, China), with each sample being replicated at least nine times. The protein, IMF, moisture, and ash contents in the LD muscle tissue were quantified using the Kjeldahl method for nitrogen determination, Soxhlet extraction technique, a drying procedure, and combustion methods. Parallel samples were replicated at least six times.

### 2.3 Non-targeted metabolomics profiling

#### 2.3.1 Metabolite extraction

Metabolite extraction was according to the method of Chen et al. (2024). The muscle tissue sample (0.1 g) was ground in liquid nitrogen and 120 μL of 50 % methanol was added for thorough mixing. The mixture was then subjected to −20 °C overnight for protein precipitation. The samples were centrifuged at 4000 rpm for 20 min and the supernatant was collected in a 96-well plate to produce quality control (QC) samples. All metabolic samples (including QC) were stored in a − 80 °C freezer before being tested.

#### 2.3.2 UPLC-MS

A UPLC system, coupled with a high-resolution mass spectrometer (Triple 5600 plus, SCIEX, UK), was used for data acquisition in both positive and negative ionization modes. Our method was based on the work of Han et al. (2021). The voltages used were 5000 V (+) and 4500 V (−), respectively. The pressure of the curtain gas in the ion source is set at 30 psi, with a temperature of 650 °C. Ion source gases 1 and 2 were set at 60 psi. The pattern was in information dependency acquisition mode and the entire collection cycle took 0.56 s. To verify and maintain data quality, the entire process involved the calibration of every 20 samples and scanning every 10 samples for QC. The experimental parameters and methods of mass spectrometry and chromatography were carried out at LC-BIO Technology Co., Ltd. Hangzhou, Zhejiang Province, China.

The UPLC chromatographic column was an ACQUITY UPLC T3 (100 mm × 2.1 mm, 1.8 μm, Waters, UK). The collection condition was the column oven was set at 35 °C and the flow rate was 0.4 mL/mL. The following additional parameters were used: Mobile phase A, water (0.1 % formic acid); mobile phase B, acetonitrile (0.1 % formic acid); gradient setting, 0–0.5 min, 5 % B; 0.5–7 min, 5 %–100 % B; 7–8 min, 100 % B; 8–8.1 min, 100 %–5 % B; 8.1–10 min, 5 % B.

### 2.4 Targeted metabolomics profiling

#### 2.4.1 Composition of amino acid

The muscle tissues of M-1, M-2, and M-3 were de-fatted and dehydrated, then dried and ground into a powder using a mortar and pestle. The sample (50 mg) was hydrolyzed with 15 mL of 6 mol/L HCl in a digestion tube at 110 °C for 22–24 h. The hydrolysate was cooled at 23 °C and mixed well, then diluted with 0.02 mol/L HCl to a final volume of 25 mL. The diluted solution (1 mL) was concentrated by drying at 60 °C, then dissolved with 0.02 mol/L HCl. After filtering through a 0.22-μm pore size filter,20 μL of the filtrate was analyzed using an amino acid analyzer (Hitachi, Tokyo, Japan).

#### 2.4.2 Composition of fatty acid

The method for extracting fatty acids from muscles was the earliest method developed by Folch et al. (1957), and modified by Zhang et al. (2024). The muscle sample (5 g) was weighed and added to a chloroform-methanol solution (2,1, 90 mL). The mixture was then extracted using a magnetic stirrer (300 rpm, 2 h). The extraction liquid was filtered and 20 % NaCl solution (5 mL) was added. The mixture was allowed to settle and the lower layer of fat extraction liquid was collected. NaOH-methanol solution (0.5 mol/L, 5 mL) was added and heated under reflux at 70 °C for 5 min to carry out a saponification reaction. Subsequently, boron trifluoride etherate (1.15 g/mL, 3 mL), n-hexane (2 mL), and saturated sodium chloride solution (5 mL) were sequentially added to obtain the esterified fatty acids. Na_2_SO_4_ powder was added for water absorption and drying. The extracted solution was passed through a nylon syringe filter.

Fatty acids were analyzed using gas chromatography (Agilent 8860, USA) fitted with Rt-2560 capillary column (100 m × 0.25 mm, 0.20 μm), helium as the carrier gas, with a flow rate of 1 mL/min, an injection temperature of 240 °C, an injection volume of 1 μL, and a split ratio of 100:1. The oven temperature program was: initial temperature of 100 °C, 5 min of stabilization; heating to 170 °C (rate: 3 °C/min), 10 min of stabilization; heating to 220 °C (rate: 3 °C/min), 5 min of stabilization; continued heating to 240 °C (rate:1 °C/min), and 10 min of stabilization. The identification of sample peaks was conducted using a mixture of 37 fatty acid methyl ester standards (Sigma-Aldrich, Darmstadt, Germany). The chromatogram of the fatty acid standard sample is shown in Supplementary Fig. 5. Quantification of fatty acids was performed according to the methodology outlined by Vahmani et al. (2017), with n-pentane serving as the internal standard. The results for individual fatty acids are expressed as mg/100 g meat.

### 2.5 Data processing and statistical analysis

Data were analyzed using one-way analysis of variance in IBM SPSS Statistics (version 22.0) to analyze carcass traits, meat quality, and fatty acid profiles. Student's two-tailed *t*-test was used for result assessment, with *P* < 0.05 considered as statistically significant. R software v3.6.3 was utilized for data analysis. Principal component analysis (PCA), orthogonal partial least squares discriminant analysis (OPLS-DA), k-means clustering analysis, and volcano map generation were conducted utilizing MetWare (https://cloud.metware.cn/). Significant variables were identified as variable for projection (VIP) from the outcomes of the OPLS-DA model. The statistical approach encompassed the application of the Benjamin–Hochberg procedure to calculate the false discovery rate (FDR), which was predicated on *P*-values. Additionally, univariate statistical methods were employed, including the assessment of fold change (FC). Volcano maps were generated based on the FC and P-value criteria. Finally, significantly different metabolites between groups were identified based on FC ≥ 2 or FC ≤ 1/2, FDR ≤ 0.05, and VIP ≥ 1 thresholds. Pathway analysis was performed via the online MetaboAnalyst 4.0 platform, and the Cytoscape software was used to visualize the regulatory network linking metabolites and pathways.

## 3 Result

### 3.1 Meat quality

**Fig. 1:**
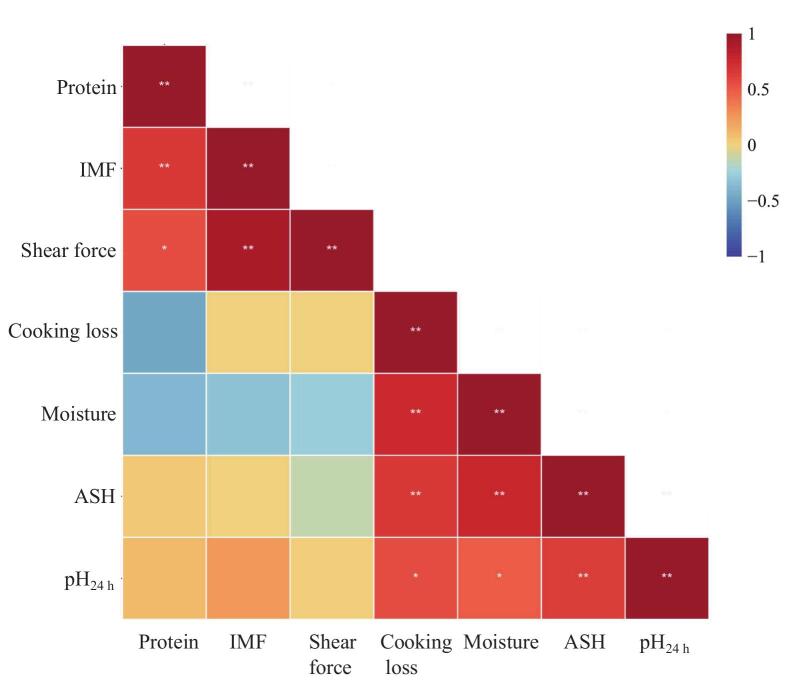
Pearson's correlation heat map of meat quality indicators in Sunit sheep. Red and blue represent positive and negative correlations, respectively; * indicates *P* < 0.05 and ** indicate *P* < 0.01. (For interpretation of the references to color in this figure legend, the reader is referred to the web version of this article.)

Table 1 illustrates the variations in meat color, shear force, cooking loss, and pH among Sunit sheep meat samples slaughtered at different ages. The L* value gradually decreases with age, and the values of M-1 and M-2 were significantly higher than those of M-3 (*P* < 0.01). The value of a* was significantly higher in M-3 than that at M-2 and M-1 (*P* < 0.01). In addition, the cooking loss of M-3 was significantly higher than those of M-1 and M-2 (*P* < 0.05). Shear force increased with age and the highest values were observed at M-3 (*P* < 0.01). The final pH_24_ value at M-3 was significantly lower than that at M-1 and M-2 (*P* < 0.001). In this study, moisture, ash, protein, and IMF were the main conventional nutrients in Sunit sheep meat (Table 1). The moisture and ash contents were significantly higher at M-1 (*P* < 0.005), compared with those at M-2 and M3, showing a decreasing trend with age. The IMF and protein contents gradually increased with age, and the IMF was significantly lowest at M-1 (*P* < 0.05). Pearson's correlation heat map of meat quality indicators is shown in Fig. 1.

**Table 1:** Physicochemical quality, texture, and color parameters of meat from sheep at different ages.

Items^1^	Different age^2^			SEM^3^	*P*-value^4^
	M-1	M-2	M-3		
L*	21.65^a^	21.56^a^	20.74^b^	0.161	0.002
a*	11.57^b^	11.30^b^	13.47^a^	0.481	0.001
b*	2.45^b^	3.28^a^	2.49^b^	0.296	0.022
Cooking loss, %	39.55^b^	39.03^b^	42.09^a^	0.010	0.050
Shear force, N	53.16^b^	58.11^b^	67.82^a^	3.274	0.005
pH_45 min_	6.43	6.54	6.41	0.077	0.252
pH_24 h_	5.37^a^	5.38^a^	5.30^b^	0.014	<0.001
Protein, %	20.90	21.13	21.44	0.003	0.310
Ash, %	1.08^a^	0.98^b^	0.96^b^	<0.001	0.005
Moisture, %	75.94^a^	74.48^b^	74.46^b^	0.003	<0.001
IMF, %	3.25^b^	4.28^a^	4.53^a^	0.004	0.017

Statistical significance was set at *P* < 0.05.

1 L*, lightness; a*, redness; b*, yellowness, IMF, intramuscular fat.

2 M-1, six-month-old; M-2, eighteen-month-old; M-3, thirty-month-old.

3 SEM, standard error of mean; *n* = 6. The values with different letters of a and b mean the value among different groups are significant different.

4 *P* -value of ANOVA indicate any significant difference between groups.

### 3.2 Non-targeted metabolomic analysis

#### 3.2.1 Multivariate statistical analysis

**Fig. 2:**
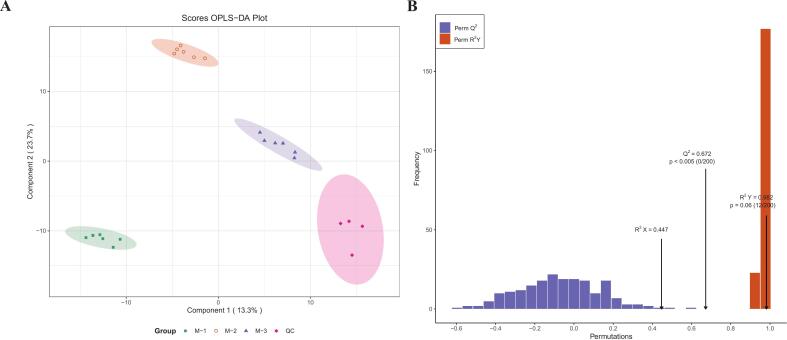
Establishment and validation of the Orthogonal partial least squares discriminant analysis (OPLS-DA) model (M-1 vs. M-2 vs. M-3, including QC). (A) Plot of OPLS-DA scores at various ages. (B) OPLS-DA model validation: the permutation test showed R^2^X = 0.447, R^2^Y = 0.982, and Q^2^ = 0.672.

In this study, muscle tissue samples from animals of different ages were analyzed using non-targeted metabolomics. To ensure comprehensive control over the signal intensity of all samples (including the QC samples), a total ion flow graph was constructed (Supplementary Fig. 1). The overlap of the graphs proved that the metabolome data had good repeatability and reliability. A total of 4318 ions were identified in the positive ion mode, while 3293 ions were identified in the negative ion mode. A combination of unsupervised PCA (Supplementary Fig. 2) and supervised OPLS-DA was used to discern the disparities in metabolites at different ages. The results showed that the prediction parameters R^2^Y = 0.982 and goodness of prediction Q^2^ = 0.672 (*P* < 0.005) for the OPLS-DA evaluation models, indicating that the model used in this study was excellent (Fig. 2A, B).

**Fig. 3:**
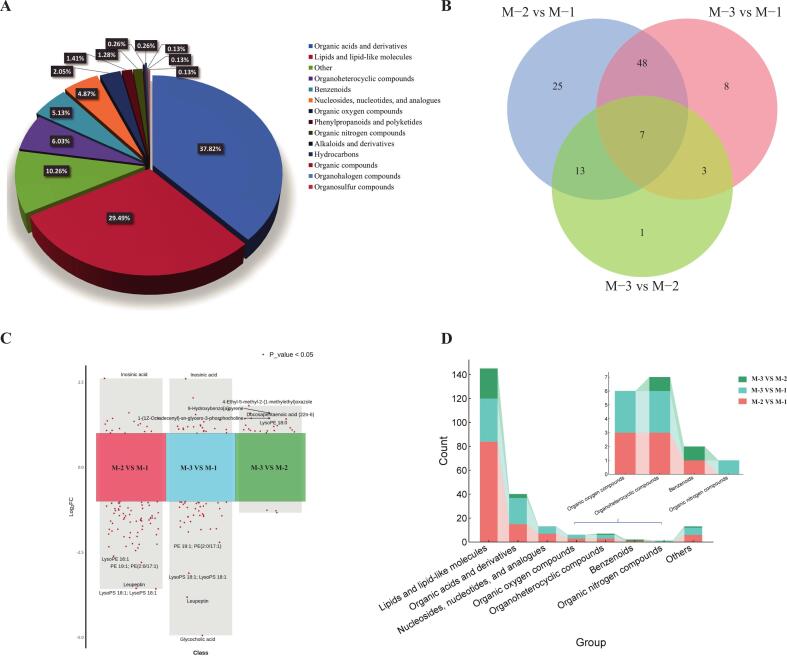
Metabolites in Sunit sheep muscle tissue identified at varying ages. (A) Pie chart illustrating the proportions of identified metabolite classes. (B) Venn diagram of muscle metabolites across three age groups. (C) Multi-group volcano plot displays the differential metabolites across various ages (M-2 vs. M-1; M-3 vs. M-1; M-3 vs. M-2). Red dots of multi-group volcano maps represent significant (*P* < 0.05). (D) Stacked bar chart showing the comparison of different metabolite classes between various ages. (For interpretation of the references to color in this figure legend, the reader is referred to the web version of this article.)

A total of 780 metabolites were detected (Supplementary Table 1) by secondary mass spectrometry and categorized into 14 subclasses, including 295 (37.82 %) organic acids and derivatives; 230 (29.49 %) lipids and lipid-like molecules; 47 (6.03 %) organoheterocyclic compounds; benzenoids 40 (5.13 %); 38 (4.87 %) nucleosides; nucleotides, and analogs; 22 (2.05 %) organic oxygen compounds; 11 (1.41 %) phenylpropanoids and polyketides; 10 (1.28 %) organic nitrogen compounds; 2 (0.26 %) alkaloids and derivatives and hydrocarbons; organic compound, organohalogen compounds, and organosulfur compounds each have one, accounting for 0.13 %; and 80 (10.26 %) other metabolites (Fig. 3A). Classifying heat maps enabled a more intuitive observation of changes in the metabolites of Sunit sheep muscle at different ages (Supplementary Fig. 3). Organic acid derivatives, lipids, and lipid-like molecules are the predominant classes of metabolites.

#### 3.2.2 Metabolic changes at different age

To better elucidate the alterations in muscle tissue metabolites of Sunit sheep at different ages, a comparative analysis was conducted among the three groups. Based on the contribution of each metabolite with a VIP ≥1.5 and FDR <0.05, in combination with univariate and multivariate statistical analysis, 105 significantly different substances were identified. The Venn Diagram revealed the presence of both shared and unique metabolites, exhibiting significant variation across different ages (Fig. 3B). The highest number of differential metabolites (93) was observed between M-2 vs M-1. Additionally, 66 and 24 differential metabolites were identified in the M-3 vs M-1 and M-3 vs M-2 comparisons, respectively. Multiple volcano plots were drawn based on the FC and *P*-values, allowing clear observation of significantly upregulated and downregulated metabolites in each comparison group (Fig. 3C). The combined bar chart classifies the 105 metabolites with significant differences into eight categories (Fig. 3D). Among these, lipids and lipid-like molecules were the main categories, Sunit sheept exhibited between M-2 vs M-1, followed by organic acids and derivatives. Overall, the metabolites with significant differences mainly converged in the comparison of M-2 vs M-1 and M-3 vs M-1.

**Fig. 4:**
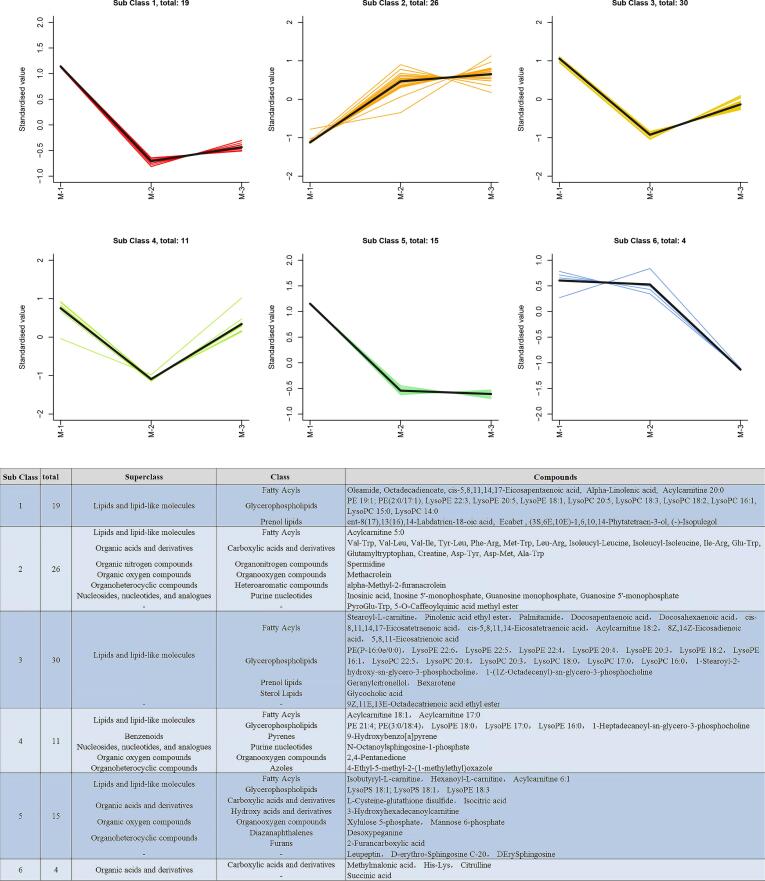
K-means clustering analysis of the distribution of differential metabolites, as well as the categorization and count of distinct metabolites within each cluster.

To comprehensively investigate the dynamic changes in the metabolites of Sunit sheep at different ages, a k-means clustering algorithm was used to analyze 105 labeled metabolites. Based on their cumulative patterns, the metabolites were categorized into six distinct clusters (Fig. 4). Overall, differential metabolites were visualized, with the fewest in subclass 2 (26 metabolites) at M-1. In the other five subclasses (comprising 79 metabolites), the metabolites were at the highest levels in M-1. Notably, with the exception of the four metabolites in subclass 6, the changes in metabolites were extremely significant for the remaining 101 metabolites at 6 and 18 months of age. Analysis of these six clusters revealed dynamic changes in metabolites at different ages.

#### 3.2.3 Metabolic pathway analysis

**Fig. 5:**
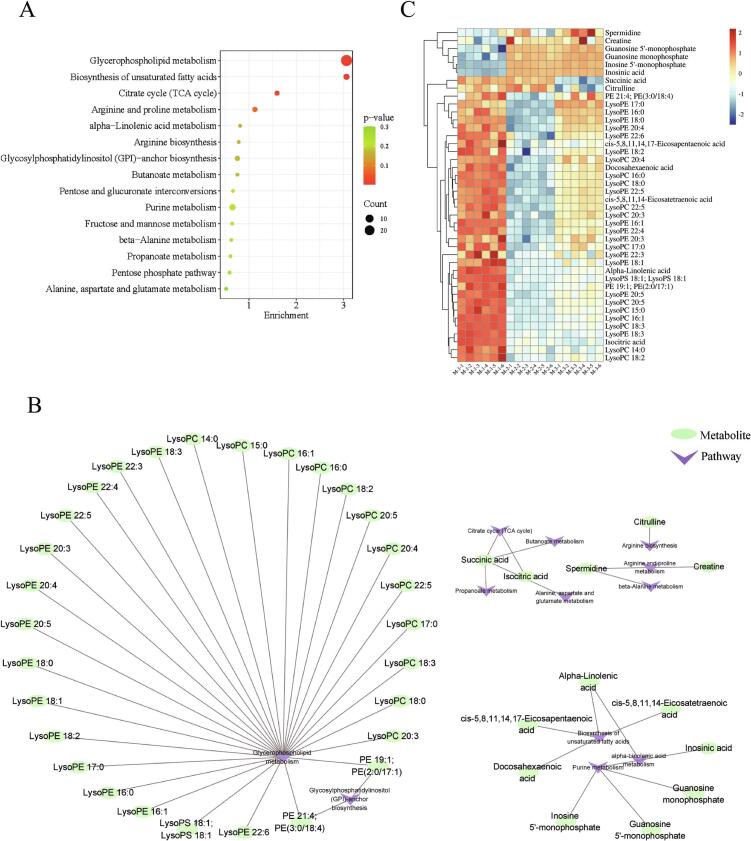
KEGG pathway analysis of differential metabolites. (A) Enrichment pathways for differential metabolites. (B) Network diagrams of enrichment pathways and annotation differential metabolites. (C) Heat map of the annotated differential metabolites of the enrichment pathways.

To highlight the various metabolic pathways of Sunit sheep muscle at different ages, KEGG enrichment was used to analyze 105 different metabolites. These differential metabolites were enriched in 23 pathways, and the top 15 major pathways were selected for analysis based on *P*-values and enrichment (Fig. 5A). The main pathways involved include glycerophospholipid metabolism, biosynthesis of unsaturated fatty acids (UFAs), the citrate cycle (TCA cycle), and arginine and proline metabolism. In addition, pathways associated with muscle metabolism in Sunit sheep include alpha-linolenic acid (ALA) metabolism; arginine biosynthesis; butanoate metabolism; glycosylphosphatidylinositol-anchor biosynthesis; pentose and glucuronate interconversions; purine metabolism; fructose and mannose metabolism; beta-alanine metabolism; propanoate metabolism; the pentose phosphate pathway; and alanine, aspartate, and glutamate metabolism. Furthermore, we constructed metabolic pathway and metabolite network diagrams (Fig. 5B), and visualized the dynamic changes in the annotated metabolites using heat maps (Fig. 5C).

### 3.3 Targeted metabolomics profiling

#### 3.3.1 Amino acid composition during different ages

Table 2 shows that the amino acid profiles of sheep muscle tissue underwent changes at different ages. Of these, the glycine, leucine, and phenylalanine contents were significantly lower at M-2 than those at M-1 and M-3 (*P* < 0.05). The cysteine content was lowest at M-1 (*P* < 0.05). Notably, the essential amino acids (EAAs), nonessential amino acids (NEAAs), and total amino acids (TAAs) contents were highest at M-3 compared with those in the other two groups, which is consistent with the observed trend of differential alterations in organic acids and derivatives within the non-targeted metabolomics analysis.

**Table 2:** Effect of different ages on the amino acid content in the muscles of Sunit sheep (mg/100 g meat).

Item^1^	Different age^2^			SEM^3^	*P*-value^4^
	M-1	M-2	M-3		
aspartic acid	30.35	31.95	32.50	1.56	0.420
threonine	16.59	16.2	16.61	0.28	0.340
serine	13.06	13.29	13.62	0.46	0.510
glutamic acid	44.75	47.59	47.81	1.82	0.250
glycine	16.07^a^	15.28^b^	16.23^a^	0.16	0.002
alanine	21.63	20.91	21.77	0.35	0.100
cysteine	2.77^b^	4.08^a^	4.35^a^	0.42	0.020
valine	17.82	17.34	18.02	0.25	0.080
methionine	10.48	9.79	10.15	0.30	0.148
isoleucine	17.75	17.15	17.66	0.28	0.150
leucine	31.86^a^	30.48^b^	31.64^a^	0.47	0.053
tyrosine	13.53	12.83	13.42	0.31	0.130
phenylalanine	16.18^a^	15.42^b^	16.18^a^	0.30	0.069
lysine	27.42	28.07	28.78	0.56	0.130
histidine	14.01	14.09	14.28	0.18	0.360
arginine	25.51	24.60	25.20	0.47	0.220
EAAs	138.09	134.45	139.04	2.33	0.195
NEAAs	181.67	184.63	189.16	4.84	0.360
TAAs	319.76	319.08	328.2	7.04	0.410

Statistical significance was set at *P* < 0.05.

1 EAAs = (leucine + methionine + valine + isoleucine + threonine + phenylalanine + lysine). EAAs: essential amino acids; NEAAs: nonessential amino acids; TAAs: total amino acids.

2 M-1, six-month-old; M-2, eighteen-month-old; M-3, thirty-month-old.

3 SEM, standard error of mean; n = 6. The values with different letters of a and b mean the value among different groups are significant different.

4 *P* -value of ANOVA indicate any significant difference between groups.

#### 3.3.2 Fatty acid composition during different ages

The impact of different ages on fatty acid composition in Sunit sheep muscle is presented in Table 3. A total of 10 saturated fatty acids (SFAs), 8 monounsaturated fatty acids (MUFAs), and 9 PUFAs were identified. The total SFA content exhibited a significant progressive increase with advancing age (*P* < 0.001). Comparative analysis revealed significantly higher concentrations of C10:0, C15:0 and C17:0 in M-3 compared to M-1 and M-2 (*P* < 0.05). Notably, C14:0 demonstrated significantly lower levels in M-1 than in M-3 (*P* < 0.05). C16:0 showed significant variations across all three groups (*P* < 0.001), with a progressive increase corresponding to advancing age. Furthermore, C18:0 concentrations were significantly lower in M-1 compared to both M-2 and M-3 (*P* < 0.05).

**Table 3:** Effect of different ages on the fatty acid content in the muscles of Sunit sheep (mg/100 g meat).

Item^1^	Slaughter age^2^			SEM^3^	*P*-value^4^
	M-1	M-2	M-3		
C10:0	3.24^b^	4.23^b^	6.11^a^	0.75	0.022
C12:0	3.69	2.94	4.35	0.73	0.237
C13:0	47.68	39.68	57.02	11.21	0.364
C14:0	49.08^b^	50.23^ab^	64.06^a^	5.80	0.075
C15:0	8.01^b^	10.03^b^	16.30^a^	1.93	0.012
C16:0	614.21^c^	787.73^b^	914.29^a^	26.36	<0.001
C17:0	28.77^b^	35.42^b^	49.27^a^	3.98	0.006
C18:0	461.06^b^	585.08^a^	607.88^a^	18.05	<0.001
C21:0	22.71	35.97	21.11	19.81	0.725
C22:0	124.05	134.55	148.09	17.09	0.424
**SFA**	1362.5^c^	1685.86^b^	1888.49^a^	53.34	0.000
C14:1	25.82	13.57	7.21	12.33	0.371
C15:1	83.55	95.43	103.32	24.72	0.735
C16:1	57.13^b^	87.36^ab^	81.26^a^	10.12	0.053
C17:1	26.82^c^	37.11^b^	46.73^a^	3.62	0.005
cis9-C18:1	1056.71^b^	1571.45^a^	1699.94^a^	71.56	<0.001
trans-9-C18:1	36.02^b^	42.93^ab^	50.47^a^	3.35	0.014
C20:1	85.57	80.16	88.47	10.47	0.735
C24:1	67.31	67.51	74.14	7.19	0.586
**MUFA**	1438.92^b^	1995.52^a^	2151.53^a^	85.31	0.000
trans-C18:2 n-6	13.86^b^	23.31^a^	25.84^a^	2.69	0.010
C18:2 n-6	271.85	260.38	308.16	27.57	0.271
gamma-C18:3 n-6	6.93	5.28	6.41	2.27	0.766
C18:3 n-3	18.10^b^	22.93^ab^	27.59^a^	2.31	0.018
C20:2 n-6	12.24	11.93	12.58	1.91	0.944
C20:3 n-3	58.38	54.94	54.68	6.84	0.838
C20:4 n-6	22.07	9.89	19.01	6.73	0.248
C20:5 n-3	12.50	8.58	11.62	3.33	0.506
C22:6 n-6	14.42	13.84	13.05	1.43	0.650
**PUFA**	430.34	411.08	478.91	35.84	0.229
**SFA/UFA**	0.73	0.70	0.72	0.04	0.823
**PUFA/SFA**	0.32^a^	0.24^b^	0.25^ab^	0.03	0.070
**n-3 PUFA**	88.98	86.46	93.88	8.92	0.713
**n-6 PUFA**	341.37	324.63	385.03	28.45	0.171
**n-6/n-3 PUFA**	3.84	3.77	4.12	0.25	0.377

Statistical significance was set at *P* < 0.05.

1 SFA: saturated fatty acids; MUFA: monounsaturated fatty acids; PUFA: polyunsaturated fatty acids; n-3 PUFA: omega-3 polyunsaturated fatty acids; n-6 PUFA: omega-6 polyunsaturated fatty acids.

2 M-1, six-month-old; M-2, eighteen-month-old; M-3, thirty-month-old.

3 SEM, standard error of mean; n = 6. The values with different letters of a, b and c mean the value among different groups are significant different.

4 *P* -value of ANOVA indicate any significant difference between groups.

The total MUFA content was significantly higher in both M-2 and M-3 compared to M-1 (*P* < 0.05). This phenomenon can be attributed to the significantly lower concentrations of C16:1 and trans-9-C18:1 in M-1 compared to M-3. Additionally, C18:1 levels were markedly reduced in M-1 compared to both M-2 and M-3 (*P* < 0.05). Notably, C17:1 demonstrated significant variations across all three experimental groups (*P* < 0.001), exhibiting a progressive increase with advancing age. The total PUFA content did not show significant variations among the three groups. However, detailed analysis revealed that trans-C18:2 levels were significantly lower in M-1 compared to both M-2 and M-3 (*P* < 0.05). Similarly, C18:3 concentrations were markedly reduced in M-1 compare to M-3 (*P* < 0.05). Furthermore, the PUFA/SFA ratio in M-1 was significantly higher than that in M-2 (*P* < 0.05), while no statistically significant difference was observed between M-1 and M-3, M-2 and M-3.

### 3.4 Correlation analyses between meat quality and non-targeted metabolomic

Phenotypic information and results from the non-targeted metabolomics study were employed in a correlation analysis to investigate the link between muscle metabolism and the meat quality of Sunit sheep at various ages. As shown in (Supplementary Fig. 4 A and B), ash and moisture content were positively correlated with those of lysoglycerophospholipids (lysoPLs), ALA, isocitric acid, cis-5,8,11,14-eicosatetraenoic acid (arachidonic acid, AA), docosahexaenoic acid (DHA) and cis-5,8,11,14,17-eicosapentaenoic acid (DPA). Meanwhile, the ash and moisture values were negatively correlated with those of creatine, guanosine 5′-monophosphate (GMP), guanosine monophosphate, inosine 5′-monophosphate (IMP) and inosinic acid. However, the IMF and shear force values negatively correlated with those of lysoPLs, succinic acid, ALA, and isocitric acid; and positively correlated with those of guanosine 5′-monophosphate, guanosine monophosphate, inosine 5′-monophosphate, and inosinic acid. Meat quality was significantly affected by the regulation of glycerophospholipid metabolism, biosynthesis of UFAs, and organic acids.

## 4 Discussion

The characteristics of meat tenderness are closely related to myofibril protein and connective tissue, which are related to water-holding capacity. In this study, the shearing force value was positively correlated with age, which is related to the structure and content of the intramuscular connective tissue (He et al., 2023). The shear force value was positively related to cooking loss (Fig. 1, *P* < 0.01), which aligns with the results reported by Bao et al. (2021), who indicated that meat cooking loss increased with animal age. IMF was also positively correlated with cooking loss (*P* < 0.01), which may be due to the loss of fat within the meat during heating, thereby increasing cooking loss (Kang et al., 2024). Owing to the lack of nutritional pressure, aging leads to the accumulation of IMF within the muscles (Table 1); therefore, cooking loss increases with age. Meat color is the intuitive indicator of consumers' desire to purchase. Lower L* values correspond to darker shades and higher a* values denote an intensified reddish tint (Kang et al., 2024), due to the increase in myoglobin levels with age. In this study, the L* value decreased with age, with the lowest L* value at M-3. This is consistent with the results of Bao et al. (2021), who demonstrated that the L* value of meat decreases with age.

As expected, numerous organic acids and derivatives were identified, which is consistent with the results of Zhang et al. (2024). Notably, there was a positive correlation between age and the majority of organic acids in the sheep muscle tissue. In addition, lipids and lipid-like molecules exhibited the highest levels in M-1, which is consistent with our previous study showing that the level of lipid metabolism in deposited fat decreases with age (He et al., 2020). Many amino acids and their residues (mainly dipeptides) were identified. Cruzen et al. (2014) reported that protein hydrolysis in the meat of mature animals is less than that in the meat of young animals post-mortem. In contrast, our results show that the protein (Table 1) and hydrolysis products (Fig. 4, subclass 2) were increased in the muscles of aged sheep, resulting in an increase in amino acid content at M-3 (Table 2). Pérez-Juan et al. (2008) found that small-molecule peptides in muscle tissues can bind volatile substances to promote flavor release. A positive correlation was identified between the dipeptide levels and age. Chen et al. (2024) indicated that the release of specific flavor compounds from mutton intensified with age. However, subtle transitions between these dipeptides and amino acids occur at any time.

The distribution of amino acids determines the synthesis of different flavor compounds, which are essential for the creation of the volatile compounds responsible for aroma, produced through the Maillard reaction and Strecker degradation processes (Zhang et al., 2024). Notably, in this study, the level of the protease inhibitor, leupeptin, was significantly higher at M-1 than at M-2 and M-3 (Fig. 3C). Leupeptin specifically inhibits the activity of serine and cysteine proteases, thereby inhibiting amino acid residues located in the active center. Serine and cysteine levels were lower in the M-1 group than in the other two groups (Table 2). Except for serine, the other amino acids, including alanine, glycine, and threonine, play an important role in the generation of sweetness in meat (Watkins et al., 2013). Glutamic and aspartic acids are commonly associated with the umami taste of meat, especially in sheep (Wang et al., 2021; Watkins et al., 2013); those two amino acids increased with the age (Table 2). The above-mentioned amino acids and dipeptides were observed within the muscle tissue of the Sunit sheep (Supplementary Table 1). In addition, IMP and GMP can effectively enhance the umami taste elicited by glutamate. In this study, the IMP and GMP levels were significantly lower at M-1 than at M-2 and M-3 (Fig. 4, subclass 2). Based on the results of the k-means analysis, it was evident that subclass 2 mainly expressed changes in the flavor profile of sheep meat at different ages.

The distribution of amino acids not only impacts meat flavor but also exerts a positive influence on tenderness. In the present study, citrulline participated in arginine biosynthesis (Fig. 5B) and was negatively correlated with shear force value (Supplementary Fig. 4B). Ruminant intestinal cells absorb glutamine and glutamic acid, and release citrulline after weaning. Subsequently, citrulline is synthesized into arginine by extracellular liver cells and organs, such as the kidneys (Wu et al., 2005). The intensity of citrulline significantly dropped at M-3 (Fig. 4, subclass 6). This may be due to the extent of citrulline synthesis were weakened in sheep with aged. Additionally, Chen et al. (2023) demonstrated that arginine can weaken the intermolecular forces between muscle fibrous proteins, resulting in decreased meat shear force value. Therefore, an increase in the level of citrulline may influence the post-mortem muscle tenderness of the Sunit sheep at M-1 and M-2. In addition, creatine is involved in the metabolism of arginine and proline (Fig. 5B). Arginine is an important upstream metabolite that promotes creatine production (Supplementary Fig. 4). Arginine, glycine, and methionine were metabolized across various organs to facilitate the synthesis of creatine (Wu et al., 2022). Xiao et al. (2019) found that the muscle tissue of athletic animals exhibited a significantly higher creatine concentration than that of non-athletic muscle tissues. Muscle tissues effectively released creatine at M-2 and M-3 (Fig. 5C), which may be because sheep exercise more with age. In addition, muscle energy metabolism and muscle performance are enhanced with age, thereby resulting in increased creatine (Xiao et al., 2019). Succinic acid concentration gradually declined throughout the sheep growth process, with a significant decrease observed at M-3 (Fig. 4, subclass 6). This may be attributed to subtle alterations in muscle fiber composition during sheep development. Succinic acid has been demonstrated to induce the remodeling of skeletal muscle fibers, thereby indirectly augmenting muscular resistance to fatigue (Wang et al., 2019). From a meat nutrition perspective, succinic acid can enhance protein synthesis and exert beneficial effects on human health (Wang et al., 2019). Additionally, lysine positively affects meat tenderness (Ramírez-Zamudio et al., 2022; Zhang et al., 2024). The metabolomics results showed that the intensity of lysine decreased with age (Supplementary Table 1). In general, the metabolites related to muscle tenderness decreased with increasing sheep age.

Slaughtering age affects the distribution of fatty acids in sheep, which influences both meat quality and dietary nutrient profiles. In this study, C16:0 and C18:0 were the predominant SFAs, which is consistent with our previous investigations of adipose depots of Sunit sheep (He et al., 2020). C18:0 has been classified as a fatty acid beneficial for human health. SFAs are an essential substance that affects meat aroma, especially that of mutton. Fatty acids are released in their free form, C17:0 is considered to be the main fatty acid involved in mutton flavor (Watkins et al., 2019). The findings of this study indicate that C17:0 exhibited significantly higher levels at M-3 than those at M-1 and M-2 (Table 3). Oleic acid (cis-9-C18:1) was identified as the predominant fatty acid, demonstrating both the highest concentration among all detected fatty acids and a significant positive correlation (*P* < 0.05) with IMF content with increasing sheep age, which aligns with Martín et al. (2023). In addition, C18:1 particularly when consumed as a meat source, has been shown to effectively reduce blood cholesterol levels and mitigate the adverse effects of SFA on human physiology (Chen & Liu, 2020).

Undeniably, the positive role of PUFA in meat is worthy of attention. In the present study, PUFA showed a significant higher level in M-1 compared to M-2 and M-3 (Fig. 4). These fatty acids serve as precursors for eicosanoid biosynthesis and modulate PPAR-γ mediated lipid metabolism (Chen et al., 2020). The result of decline in PUFA levels with age-dependent may be attributed to rumen microbial β-oxidation activity increased with sheep maturity. Specifically, the changes of endogenous rumen microbes lead to the biohydrogenation of PUFA (Mapiye et al., 2012). ALA and linoleic acid (LA) are essential fatty acids. In mammals, ALA and LA are converted into long-chain ω-3 fatty acids through desaturation and elongation of the carbon chain, including EPA (20,5n-3), DPA (22,5n-3), and DHA (22,6n-3), and long-chain ω-6 fatty acids, such as AA (20,4n-6). PUFAs have been extensively investigated in the field of nutrition owing to their beneficial effects on human health and well-being (Chen et al., 2020). The expressions of ALA, EPA, DPA, DHA, and AA were highest at M-1 (Fig. 4). The high expression levels of those PUFAs at M-1 have likely reflected the enhancing δ-5 and δ-6 desaturase activity in young sheep muscle tissue, which catalyze the conversion of ALA to EPA/DHA (EC 1.14.19.3). Meanwhile, sheep undergo high lipid metabolism in the early growth stage, leading to an increase in fatty acid deposition (He et al., 2020). In addition, it augments fatty acid deposition during grazing years (Wang et al., 2021; Zhang et al., 2022). Generally, the PUFA/SFA ratio serves as a pivotal indicator for evaluating the dietary impact on cardiovascular diseases, with recommended nutritional values for lamb meat ranging from 0.13 to 0.37 (Chen et al., 2020). The PUFA/SFA ratios of the three groups were within this range. Moreover, a balanced diet containing n-6/n-3 PUFAs plays an important role in regulating the physiological functions of the human body. In general, it exhibits a more favorable distribution of fatty acids for human health.

Carnitine receives fatty acyl from acyl-CoA to produce acylcarnitine. Carnitine derivative levels in sheep meat from the grazing group were higher than those from the indoor feeding regimes (Wang et al., 2021). The carnitine derivatives play beneficial roles in several human metabolic diseases. The majority of acylcarnitines showed a statistically significant peak at M-1 in the naturally grazing Sunit sheep muscle (Fig. 4, subclass 3&4). In general, acylcarnitine serves as a transporter, delivering fatty acid derivatives into the mitochondrial matrix for the process of oxidative phosphorylation and participates in the thermogenic pathway (Zhang et al., 2024). The high-level lipid metabolism exhibited in 6-month-old sheep than that in 18- and 30-month-old sheep (Chen et al., 2024; He et al., 2020) leads to mitochondrial dysfunction and impairs the β-oxidation of fatty acids (Martín et al., 2023). However, long-chain acylcarnitines can rapidly activate muscle cell stress when cells have impaired long-chain fatty acid oxidation. This process results the accumulation of acylcarnitine and obstructs long-chain fatty acid oxidation (McCoin et al., 2015), resulting in the deposition of long-chain fatty acids. Therefore, the intensity of acylcarnitine was positively correlated with PUFA levels, which was responsible for the high relative content of lipids and PUFA metabolism at M-1 (Fig. 4, subclass 1&3). The TCA cycle is an important energy production pathway in mitochondria, and a common oxidative pathway for the metabolism of fatty and amino acids. Succinic and isocitric acid, which serve as crucial intermediates, were significantly enriched in the TCA cycle (Fig. 5B). Notably, their relative intensity reached its peak at M-1 (Fig. 5C). This may be generated by involvement of succinic and isocitric dehydrogenase. This metabolic signature implies that the TCA cycle is high activity effect in the muscle of young sheep, generating higher energy metabolism.

Phospholipids, a type of functional polar lipids, are essential components of cell membranes and are universally present in all tissues. Phospholipids in meat contain large amounts of long-chain PUFAs, which are prone to lipid oxidation during the cooking process, thereby promoting the formation of aroma compounds (Resconi et al., 2013). Furthermore, from a histological perspective, type I muscle fibers have a higher concentration of phospholipids, which is negatively correlated with shear force, thereby enhancing muscle tenderness (Zhang et al., 2024). The highest meat tenderness was exhibited by M-1 sheep because type I muscle fibers comprise a higher proportion at 6 months old (Siqin et al., 2017) than at 18 and 30 months. Meanwhile, a higher level of phospholipids was metabolized in sheep muscle at M-1 than in the other two groups. Previous studies have shown that PUFAs combined with phospholipids are more beneficial than PUFAs alone (Lordan et al., 2020). PC, PE, lysoPL, and plasmenyl-PL are involved in Sunit sheep muscle metabolism and are rich in long-chain PUFAs (Supplementary Table 1). Of these, lysoPL was significantly enriched in the glycerophospholipid metabolism pathway (Fig. 5B). During muscle metabolism, the interconversion between PL and lysoPL occurs via the glycerophospholipid metabolic pathway. In addition, lysoPL plays a pivotal role in the regulation of mammalian physiology, development, and disease; for instance, it is involved in mast cell activation (Blaho & Hla, 2011). The relative intensity of lysoPLs was high at M-1 (Fig. 5C). This high level of lysoPL may be increased by phospholipase activity during the early development of Sunit sheep, which induces the accumulation of lysoPL to activate the development of early adipocytes, thereby promoting the activation of lipid metabolism at M-1. In addition, lysoPLs can be used as supplements in functional foods that help regulate the overall and functional homeostasis of the gastrointestinal mucosa to promote human health (Tokumura, 2011). Therefore, considering both meat quality and nutritional needs, naturally grazed Sunit sheep meat is a more favorable diet. We encourage further in-depth research based on this study, such as comparisons of different feeding regimes, to explore how these metabolites involved in the process of improving meat quality.

## 5 Conclusions

Overall, 105 differential metabolites were identified in the Sunit sheep muscle at various ages (6, 18, and 30 months). Organic acids and their derivatives, lipids and lipid-like molecules were the main categories. These differential metabolites are primarily involved in the biosynthesis of unsaturated fatty acids, glycerophospholipid metabolism, TCA cycle, arginine and proline metabolism. Notably, flavor-related metabolites, including dipeptides, IMP, and GMP were found to be upregulated at M-3. Tenderness-related metabolites, such as lysoPLs, citrulline, and succinic acid were upregulated at M-1. Additionally, the accumulation of long-chain acylcarnitines was suggested a potential influence factor on the deposition of PUFAs, likely mediated through thermogenic pathways in muscle metabolism. The present research provides innovative theoretical viewpoints on the alterations in muscle metabolism and the associated metabolic pathways in naturally grazed Sunit sheep at varying ages, offering a valuable reference for both meat producers and consumers.

## CRediT authorship contribution statement

**Lu Chen:** Writing – review & editing, Writing – original draft, Visualization, Validation, Software, Methodology, Formal analysis, Conceptualization. **Xige He:** Funding acquisition, Validation, Investigation, Project administration. **Yunfei Han:** Software, Methodology. **Yajuan Huang:** Data curation, Conceptualization. **Jin Li:** Software, Investigation. **Xueting Yu:** Software, Methodology. **Xueyan Yun:** Visualization, Software. **Yirgejim Wuqier:** Software, Data curation. **Gerelt Borjigin:** Visualization, Supervision, Funding acquisition.

## Declaration of competing interest

The authors declare that they have no known competing financial interests or personal relationships that could have appeared to influence the work reported in this paper.

## Data Availability

Data will be made available on request.

## Appendix A Supplementary data

Supplementary material 1 Total ion flow graph. Image 1

Supplementary material 2 Principal component analysis. Image 2

Supplementary material 3 Heat map. Image 3

Supplementary material 4 Correlation heatmap. Image 4

Supplementary material 5 The chromatogram of the fatty acid standard sample. Image 5

Supplementary material 6 A total of 780 metabolites by secondary mass spectrometry. Image 6
