# Supplementary figures and images for: Reveals meat quality and muscle metabolism characteristics in naturally grazed Sunit sheep at different ages
Source: Food Chem X. 2025 Sep 7;31:103012. doi: 10.1016/j.fochx.2025.103012 (PMC12475852; doi:10.1016/j.fochx.2025.103012)

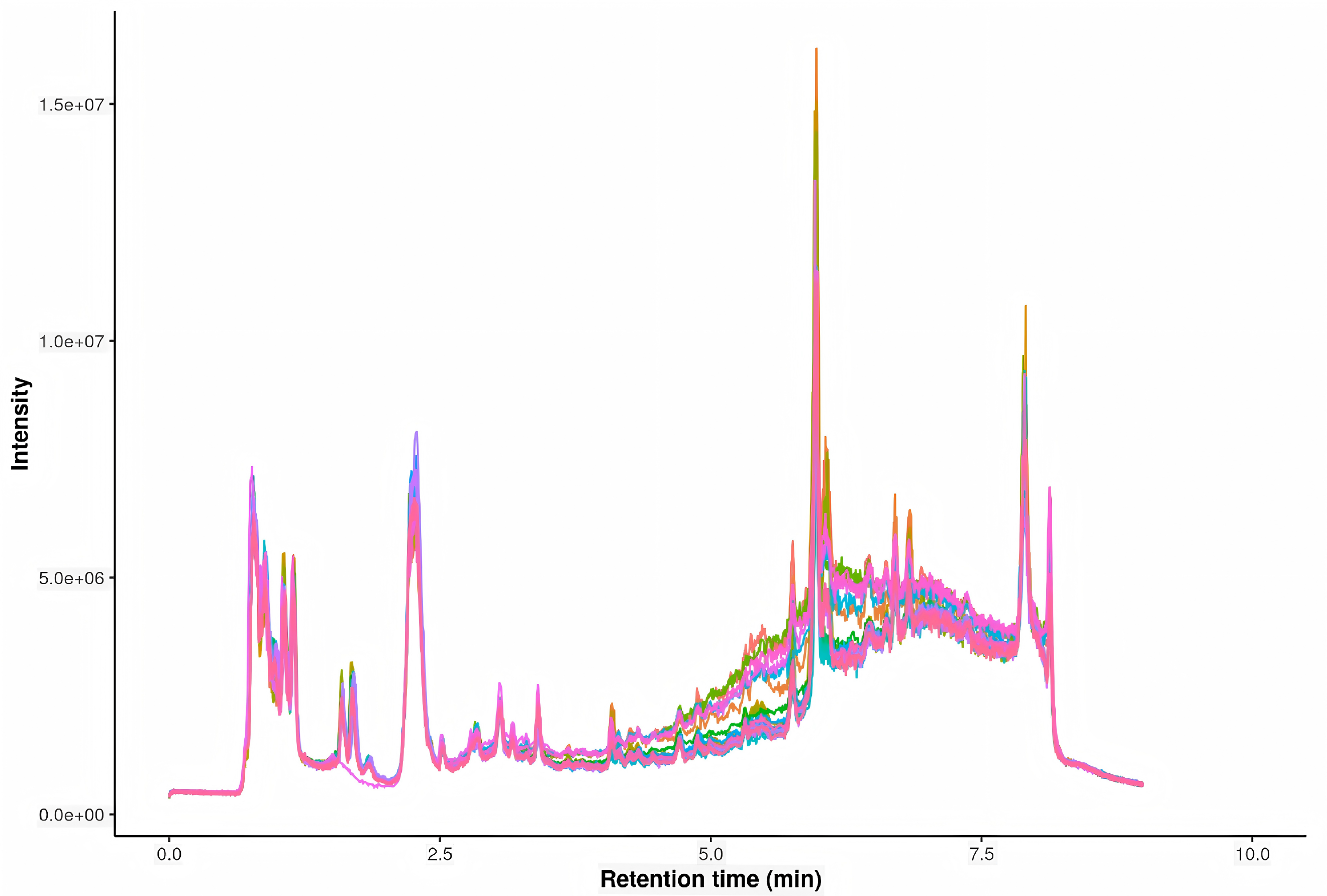

Supplement: Supplementary material 1 — Total ion flow graph. [file mmc1.pdf]

# PCA Analysis

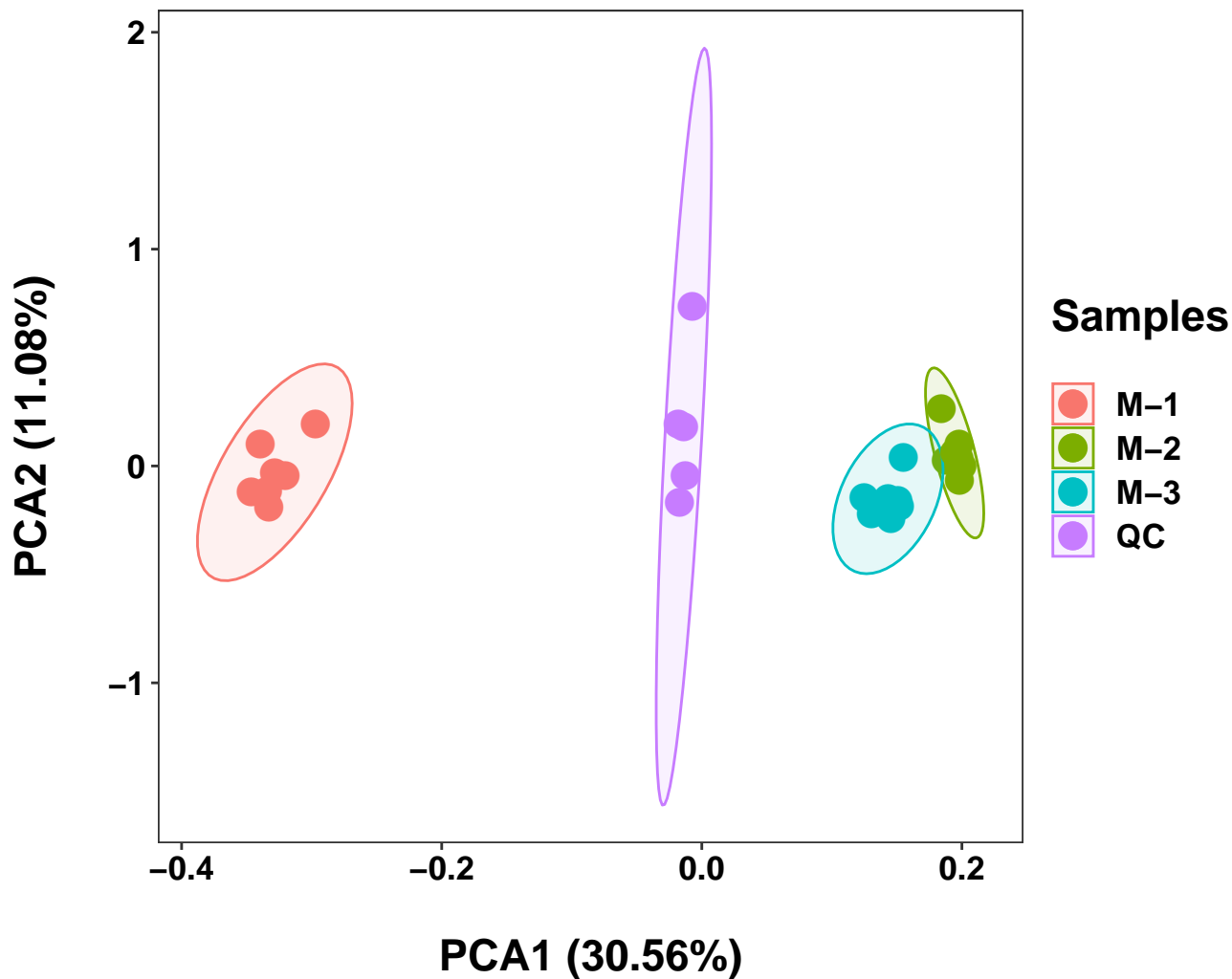

Supplement: Supplementary material 2 — Principal component analysis. [file mmc2.pdf]

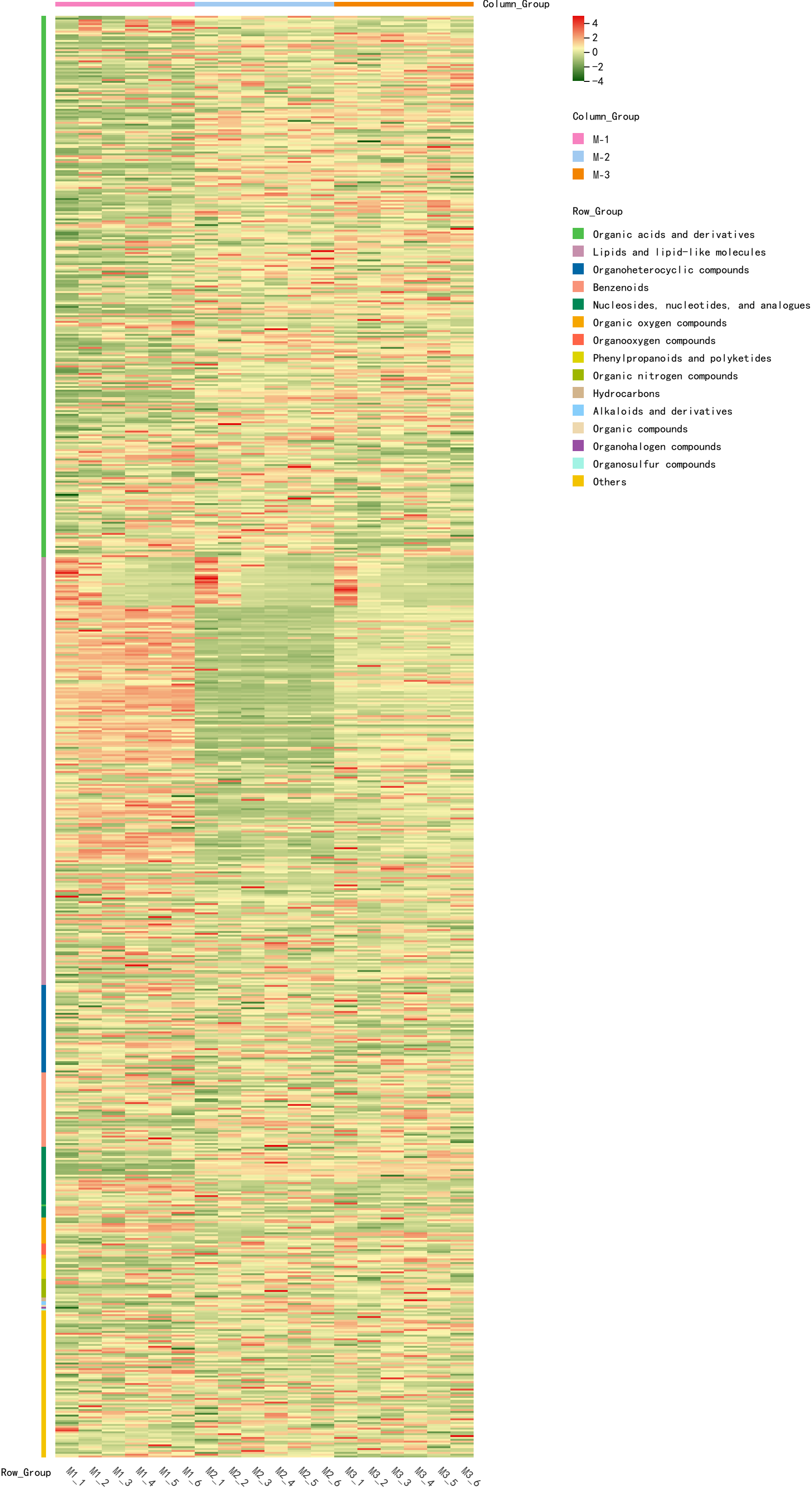

Supplement: Supplementary material 3 — Heat map. [file mmc3.pdf]

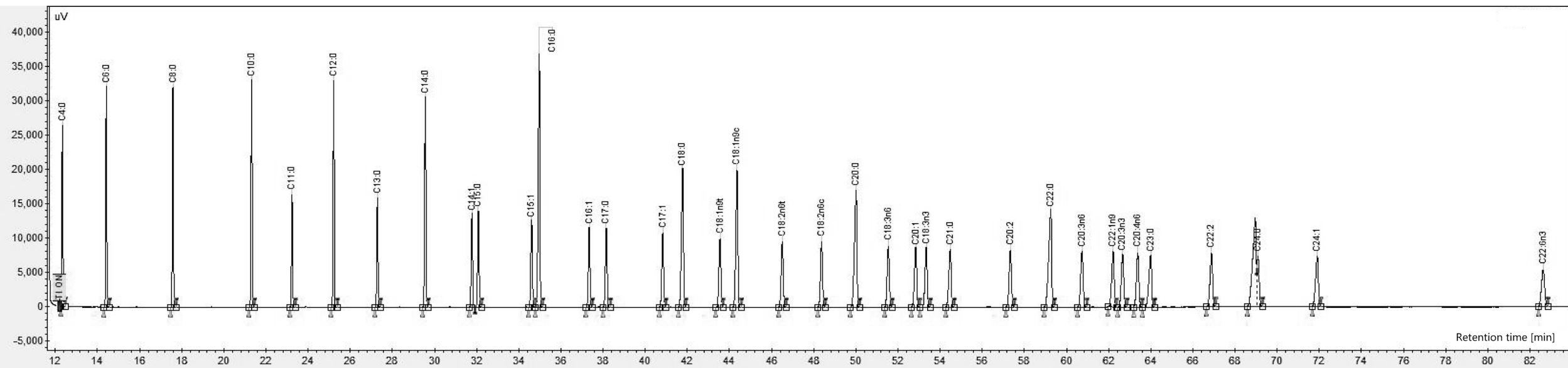

Supplement: Supplementary material 5 — The chromatogram of the fatty acid standard sample. [file mmc5.pdf]
