# Supplementary material for: Reveals meat quality and muscle metabolism characteristics in naturally grazed Sunit sheep at different ages
Source: Food Chem X. 2025 Sep 7;31:103012. doi: 10.1016/j.fochx.2025.103012 (PMC12475852; doi:10.1016/j.fochx.2025.103012)
Supplement: Supplementary material 4 — Correlation heatmap. [file mmc4.pdf]

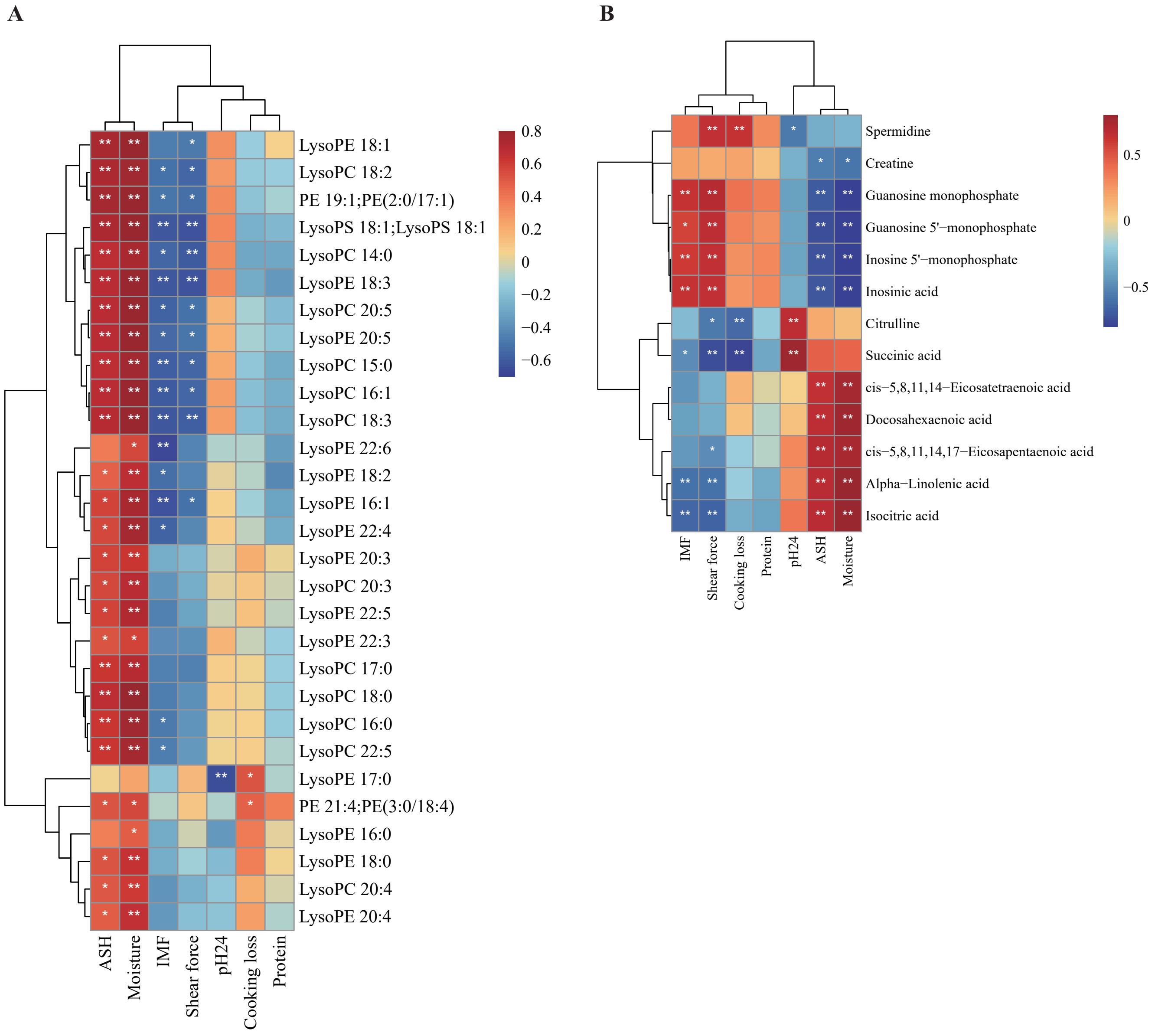
A
LysoPE 18:1
LysoPC 18:2
PE 19:1;PE(2:0/17:1)
LysoPS 18:1;LysoPS 18:1
LysoPC 14:0
LysoPE 18:3
LysoPC 20:5
LysoPE 20:5
LysoPC 15:0
LysoPC 16:1
LysoPC 18:3
LysoPE 22:6
LysoPE 18:2
LysoPE 16:1
LysoPE 22:4
LysoPE 20:3
LysoPC 20:3
LysoPE 22:5
LysoPE 22:3
LysoPC 17:0
LysoPC 18:0
LysoPC 16:0
LysoPC 22:5
LysoPE 17:0
PE 21:4;PE(3:0/18:4)
LysoPE 16:0
LysoPE 18:0
LysoPC 20:4
LysoPE 20:4
ASH
Moisture
IMF
Shear force
pH24
Cooking loss
Protein
0.8
0.6
0.4
0.2
0
−0.2
−0.4
−0.6
B
Spermidine
Creatine
Guanosine monophosphate
Guanosine 5'−monophosphate
Inosine 5'−monophosphate
Inosinic acid
Citrulline
Succinic acid
cis−5,8,11,14−Eicosatetraenoic acid
Docosahexaenoic acid
cis−5,8,11,14,17−Eicosapentaenoic acid
Alpha−Linolenic acid
Isocitric acid
IMF
Shear force
Cooking loss
Protein
pH24
ASH
Moisture
0.5
0
−0.5
